# An Intelligent IoT-Based Predictive Control System for Water Quality and Energy Management in Koi Aquaculture

**DOI:** 10.3390/s26103238

**Published:** 2026-05-20

**Authors:** Kunyanuth Kularbphettong, Nutthapat Kaewrattanapat, Nareenart Raksuntorn

**Affiliations:** 1 Computer Science Program, Faculty of Science and Technology, Suan Sunandha Rajabhat University, Bangkok 10300, Thailand; nareenart.ra@ssru.ac.th; 2 Digital Technology for Education Program, Faculty of Education, Suan Sunandha Rajabhat University, Bangkok 10300, Thailand; nutthapat.ke@ssru.ac.th

**Keywords:** smart aquaculture, IoT-based sensing, predictive control, Digital Twin, energy-aware management, LSTM, Cyber-Physical Systems

## Abstract

**Highlights:**

**What are the main findings?**

- An integrated IoT–Digital Twin–CPS framework with edge-based LSTM enables closed-loop predictive control in ornamental aquaculture.
- The proposed system achieves a 26.86% reduction in energy consumption by exploiting predictive timing and natural environmental dynamics during a 45-day real-world deployment.

**What are the implications of the main findings?**

- Demonstrates that Digital Twin-assisted validation can bridge the gap between AI prediction and safe control execution, improving system explainability and reliability.
- Provides a scalable and energy-aware aquaculture framework applicable to resource-constrained and small-scale smart farming environments.

**Abstract:**

Reducing energy consumption while maintaining stable water quality remains a major challenge in ornamental aquaculture. This study proposes an integrated predictive and energy-aware aquaculture management framework combining Internet of Things (IoT) sensing, Long Short-Term Memory (LSTM)-based prediction, Digital Twin (DT) simulation, and Cyber-Physical System (CPS) control. Real-time sensor networks monitored dissolved oxygen (DO), ammonia (NH_3_), temperature, pH, turbidity, and energy consumption in a koi pond over a 45-day deployment period. Forecasted environmental states generated by the LSTM model were validated through a physics-informed Digital Twin prior to actuator execution to improve operational reliability and control safety. Experimental results demonstrated strong agreement between the Digital Twin and observed pond dynamics, achieving R^2^ values of 0.97 for dissolved oxygen and 0.94 for ammonia. Compared with conventional manual operation, the proposed smart predictive control mode reduced total energy consumption by 26.86%. Statistical analysis confirmed that the reduction was highly significant (*p* < 0.001), with average daily energy consumption decreasing from 212 ± 6.06 Wh/day under manual operation to 154.71 ± 4.52 Wh/day under smart predictive control.

## 1. Introduction

Aquaculture has become an essential component of global food systems in response to increasing demand for sustainable protein sources. In addition to food production, ornamental aquaculture, particularly koi (*Cyprinus carpio*), plays a significant economic and cultural role in many regions. Koi aquaculture, despite its significance, remains predominantly governed by manual observation and experiential methodologies and such methodologies frequently result in ineffective resource allocation, volatile environmental conditions, and heightened operating expenses. Fluctuations in essential water quality parameters, such as dissolved oxygen (DO), ammonia (NH_3_), temperature, and pH, directly impact fish health and system stability, while also affecting the energy requirements for aeration, feeding, and water circulation operations [1,2].

Recent advances in the Internet of Things (IoT), Cyber-Physical Systems (CPS), and data-driven intelligence have facilitated real-time monitoring and automated control in aquaculture settings [1,3]. IoT-based sensing networks offer continuous environmental monitoring, whereas CPS-oriented designs amalgamate sensing, computation, and actuation to facilitate adaptive system responses [3,4] and deep learning techniques have significantly improved the capacity to forecast short-term environmental changes and facilitate informed decision-making in aquaculture systems [1,5]. In numerous existing systems, prediction outputs are utilized to initiate control actions without intermediary validation, thereby introducing operational hazards in uncertain or rapidly changing settings [1,5].

Digital Twin (DT) technology has emerged as a promising approach to bridge this gap by enabling virtual representations of physical systems for monitoring, simulation, and scenario evaluation [5,6]. Nevertheless, current DT implementations in aquaculture are often limited to data synchronization, architecture design, or visualization-oriented platforms, with limited incorporation of physically interpretable models and systematic validation in real pond environments [4,5]. As a result, their role in supporting operational decision-making remains constrained. In particular, the distinction between prediction and simulation is often unclear: predictive models estimate future states, whereas a Digital Twin can evaluate the consequences of potential control actions under simplified physical and biological constraints [5,6].

The integration of these technologies is conceptually aligned with the Precision Fish Farming (PFF) paradigm, which emphasizes continuous sensing, biological-state interpretation, and adaptive management in aquaculture [2]. Related developments in controlled-environment agriculture have shown that integrating sensing, prediction, and automation can improve environmental stability and operational efficiency in small-scale production systems [7]. However, applying similar principles to ornamental aquaculture is more complex because aquatic systems involve water-borne dynamics, fish physiological responses, and energy-intensive operations that must be managed simultaneously [2,7].

Another important limitation in existing smart aquaculture systems is the insufficient consideration of energy consumption as an explicit control objective. Most prior studies focus primarily on environmental monitoring accuracy or production efficiency, while energy usage is treated as a secondary outcome [1,3]. In practice, however, energy-intensive operations, especially aeration, feeding, and water circulation, constitute a major portion of operational costs. Conventional control strategies often rely on fixed schedules or reactive thresholds, leading to unnecessary energy consumption, particularly when actuators operate continuously or excessively despite acceptable environmental conditions [1].

To address these limitations, this study proposes an integrated closed-loop aquaculture management framework that explicitly links environmental prediction, simulation-based validation, and energy-aware control. Unlike conventional approaches that rely solely on reactive thresholds or direct prediction-to-action mapping, the proposed framework incorporates a Digital Twin component as a decision-validation layer. This DT component integrates simplified physics-informed relationships, such as oxygen transfer behavior and ammonia accumulation, with real-time sensor data, enabling scenario-based evaluation of candidate control actions before physical implementation [5,6]. In addition, the framework employs a multi-rate data processing strategy: high-frequency sensing at 1-min intervals supports rapid anomaly detection, while 5-min resampled data are used for deep learning and simulation tasks to reduce stochastic noise and improve computational stability.

Within this framework, short-term forecasts generated by a Long Short-Term Memory (LSTM) model are not directly executed as control commands but are first evaluated within the Digital Twin environment to assess potential impacts on water quality and system stability. This design helps distinguish between “what is likely to happen” and “what may happen if a specific control action is applied,” thereby reducing the risk of inappropriate actuation in dynamically changing pond environments [5,6].

A key focus of this study is the energy–water quality trade-off in ornamental aquaculture. The proposed approach integrates energy consumption as a distinct control variable rather than considering it a mere consequence and the system utilizes predictive insights and simulation-based validation to facilitate proactive aeration management, preventing continuous or excessive operation while environmental conditions are within safe parameters. The framework seeks to stabilize the habitat microclimate and mitigate physiological stress caused by fast environmental variations in manual pond management by keeping DO and NH_3_ within physiologically acceptable limits [1,2].

The contributions of this study are threefold. First, it presents an integrated IoT–DT–CPS framework that combines sensing, prediction, simulation, and control within a unified closed-loop architecture for ornamental aquaculture [1,3,4,5,6]. Second, it provides an empirical evaluation of predictive control under pilot-scale real-world koi pond conditions over a 45-day deployment period. Third, it analyzes the interaction between environmental stability and energy consumption under both manual and automated operation modes. Rather than introducing a fundamentally new architecture, this work provides a systematic and experimentally grounded assessment of how Digital Twin-assisted control can support energy-aware aquaculture management in small-scale koi farming systems.

## 2. Related Work

The integration of sensing, computation, and control technologies has been extensively explored in smart aquaculture and related domains. Existing studies can be broadly categorized into five research streams: IoT-based environmental monitoring, Digital Twin (DT) modeling, Cyber-Physical Systems (CPS), deep learning-based predictive analytics, and energy monitoring and optimization. This section critically reviews these domains and identifies the limitations that motivate the present study.

### 2.1. IoT-Based Monitoring in Aquaculture Systems

The Internet of Things (IoT) has become a foundational technology for real-time environmental monitoring across various domains, supported by advances in connectivity, scalability, security, and system reliability [8,9,10,11,12,13,14]. Also, in aquaculture, IoT-based systems have been widely adopted to monitor key environmental parameters such as temperature, dissolved oxygen, pH, ammonia, and turbidity, thereby improving situational awareness and operational transparency [15,16,17,18,19,20].

Several studies have demonstrated the practical value of IoT-enabled aquaculture platforms. For example, cloud-based monitoring systems have improved measurement accuracy and reduced labor requirements through automated data acquisition and remote management [18]. Intelligent aquaculture systems featuring automated feeding and aeration have been shown to enhance operational efficiency and survival rates [19,20]. IoT-based sensing frameworks have been effectively utilized in precision agriculture to facilitate data-driven environmental monitoring and adaptive farm management [21,22,23,24]. Analyses of intelligent aquaculture have also underscored the facilitative function of IoT, cloud computing, and artificial intelligence in the modernization of aquatic production systems [1].

The majority of IoT-based aquaculture systems are still focused on data acquisition, transmission, and visualization, with only a limited integration of adaptive control or predictive analytics, despite these advancements and consequently, numerous systems operate mostly as monitoring platforms instead of closed-loop decision-support systems. Unresolved challenges persist regarding resilience, energy efficiency, and deployment reliability in dynamic aquatic environments.

### 2.2. Digital Twin-Based Modeling in Aquaculture

Digital Twin (DT) technology extends IoT capabilities by enabling virtual representations of physical systems for monitoring, simulation, prediction, and optimization [2,25,26,27,28,29,30]. In industrial and manufacturing contexts, DT research has increasingly emphasized model structure, synchronization mechanisms, and the use of virtual counterparts for predictive analysis and system-level optimization [25,26,29]. These studies show that the value of a DT depends not only on real-time data linkage but also on the interpretability and validity of the underlying model.

In aquaculture, DT concepts have only recently begun to emerge. Lan et al. [4] and Ubina et al. [5] proposed DT-oriented architectures for intelligent fish farm management, demonstrating the potential of IoT-enabled integration in aquatic systems. However, these studies are primarily oriented toward architecture design, data integration, and intelligent monitoring. A substantial gap remains in the incorporation of physically interpretable process representations, such as oxygen transfer and nutrient dynamics, which are necessary for scenario-based validation of control actions before physical execution.

More generally, many DT implementations in aquaculture remain conceptual, data-driven, or visualization-oriented, with limited attention to parameter calibration and validation against real pond behavior. In practice, this means that the DT is often used to mirror the physical system rather than to rigorously evaluate candidate operational decisions. For aquaculture control applications, however, this distinction is critical: a predictive model estimates what is likely to happen, whereas a DT should also help assess what may happen if a specific action is applied under physical and biological constraints. This limitation highlights the need for hybrid DT approaches that integrate real-time sensing with simplified but interpretable process relationships and calibration-aware validation procedures.

### 2.3. Cyber-Physical Systems for Adaptive Control

Cyber-Physical Systems (CPS) offer a framework for the integration of sensing, computation, communication, and actuation within closed-loop control frameworks and CPS has been extensively utilized in industrial automation, smart manufacturing, and intelligent infrastructure to facilitate real-time monitoring and adaptive system functionality [28]. In agriculture, CPS concepts have been further applied to smart irrigation and automated resource management [31].

CPS-based methodologies have been suggested in aquaculture for the automation of feeding, aeration, and pond management via real-time sensing and rule-based decision-making [30,32], and these technologies diminish manual intervention and enhance operational responsiveness. Nonetheless, the majority of existing solutions are predominantly reactive, meaning that actions are initiated solely after environmental thresholds have been approached or surpassed.

From the perspective of Precision Fish Farming (PFF), this represents only a partial realization of the broader vision of continuous monitoring, biological-state interpretation, and adaptive decision support [2]. A more complete CPS implementation for aquaculture should not only sense and react, but also integrate prediction and simulation into the actuation pathway. This is particularly important in ornamental aquaculture, where water quality disturbances may develop rapidly and proactive intervention is preferable to threshold-based correction.

### 2.4. Deep Learning for Predictive Analytics

Deep learning and machine learning methodologies have been progressively utilized in aquaculture for functions including water quality forecasting, fish behavior assessment, biomass estimation, and health monitoring [33,34], and these methods are particularly attractive because they can capture nonlinear temporal patterns and complex relationships in environmental and biological data.

Several studies have demonstrated strong predictive accuracy using neural-network-based models integrated with IoT platforms [15,35]. Recurrent architectures, including LSTM and similar deep learning models, have demonstrated potential for predicting time-varying water quality metrics and facilitating intelligent aquaculture applications [33,35]. In comparison to conventional statistical methods, these models provide benefits in temporal feature extraction and nonlinear modeling capabilities.

However, most existing deep learning applications function as standalone predictive modules. Their primary contribution is typically reported in terms of forecasting accuracy, such as low RMSE or MAE values, without examining how prediction outputs are used in downstream control systems. As a result, there remains limited understanding of how prediction errors propagate through automated decision layers and subsequently affect environmental stability, fish welfare, or system energy overhead. This disconnect between predictive performance and operational consequence represents an important methodological gap in smart aquaculture research.

### 2.5. Energy Monitoring and Optimization

Energy consumption is a critical concern in IoT-enabled systems and aquaculture operations, particularly due to energy-intensive processes such as aeration, water circulation, and feeding and prior research has examined energy monitoring, smart metering, and low-power design within IoT contexts [36,37,38,39,40]. These initiatives have enhanced transparency regarding power consumption and facilitated the advancement of more efficient embedded devices and monitoring systems.

Nevertheless, the majority of current research focuses on energy largely via monitoring, hardware efficiency, or static duty-cycling methodologies [36,40]. For example, IoT-based energy monitoring platforms have been widely used to track real-time consumption in residential and general-purpose settings [37,38,39], but such approaches do not directly address how energy usage can be reduced through intelligent, environment-aware control decisions. In aquaculture, this distinction is particularly important because actuator demand is tightly coupled to biological and environmental conditions rather than fixed operating schedules.

Accordingly, energy-aware predictive control remains underexplored in aquaculture, especially in ornamental fish farming. Existing literature rarely treats energy consumption as a primary real-time control objective, and few studies quantify the extent to which energy reduction can be attributed specifically to predictive intelligence rather than to hardware changes or preset duty cycles. This gap motivates the present study, which focuses on proactive, simulation-validated actuation logic as the basis for reducing energy use.

### 2.6. Research Gap and Positioning

Based on the above review, the following research gaps can be identified.

First, there is a lack of intermediate validation layers in most smart aquaculture systems. Existing approaches often map prediction directly to action, with little or no simulation-based verification of whether a candidate control response is biologically and operationally appropriate.

Second, Digital Twin implementations in aquaculture generally show insufficient physics integration. Many systems emphasize data synchronization and interface design but lack physically interpretable structures, such as oxygen transfer or nutrient–dynamics relationships, that are necessary for reliable scenario testing and model-based control support.

Third, the objectives of predictive control and energy monitoring are not in alignment. Although energy use is recognized as an important operational issue, it is rarely incorporated as a primary, real-time control objective in ornamental fish farming. Most prior studies either monitor power consumption or reduce energy through static operating rules, rather than through forecast-informed actuation.

To address these gaps, this study proposes an integrated IoT–DT–CPS–DL framework for ornamental aquaculture that combines real-time sensing, deep learning-based forecasting, physics-aware Digital Twin simulation, and closed-loop control. Unlike prior work that focuses on isolated components, the proposed framework introduces a Digital Twin-assisted validation layer between prediction and actuation, while explicitly treating energy efficiency as a control objective alongside environmental stability. In this way, the study provides an experimentally evaluated approach to proactive and energy-aware aquaculture management under real-world deployment conditions.

## 3. Proposed Methodology

This research presents a comprehensive cyber-physical aquaculture platform for energy-efficient koi pond management. The framework integrates Internet of Things (IoT) sensing, Digital Twin (DT)-facilitated state modeling, deep learning-driven prediction, and closed-loop Cyber-Physical System (CPS) control. The principal aim is to sustain biologically acceptable water quality while reducing superfluous energy expenditure linked to aeration and feeding processes. 

The sensing system consisted of a dissolved oxygen sensor (SEN0237, DFRobot, Shanghai, China), pH sensor (SEN0161-V2, DFRobot, Shanghai, China), DS18B20 temperature sensor (Maxim Integrated, San Jose, CA, USA), turbidity sensor (SEN0189, DFRobot, Shanghai, China), MQ-137 ammonia sensor (Winsen Electronics, Zhengzhou, China), and PZEM-004T energy meter (Peacefair, Shenzhen, China). The software architecture was developed using Arduino IDE 2.3 and Python 3.11 and related statistical libraries.

**Figure 1:**
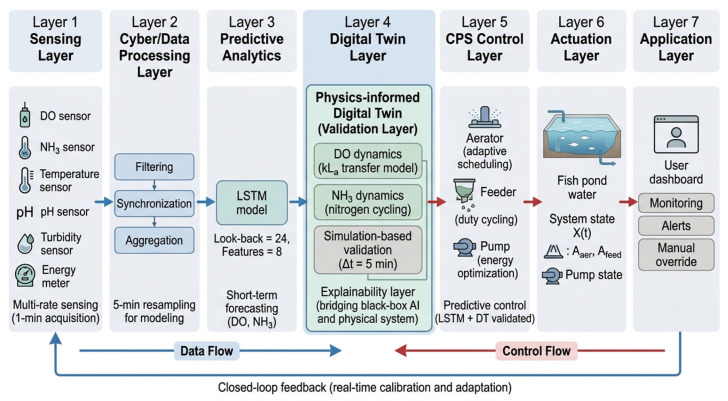
An IoT-DT System Framework.

To ensure methodological transparency, the system is structured into six interconnected components: (i) sensing and data acquisition, (ii) data preprocessing and synchronization, (iii) predictive modeling, (iv) Digital Twin-based simulation and validation, (v) CPS-based control decision-making, and (vi) feedback-driven adaptation. Figure 1 illustrates the architecture and the interaction between data flow and control flow.

### 3.1. IoT–DT–CPS System Framework

The proposed system is formulated as a closed-loop cyber-physical architecture linking the physical pond, the cyber layer, and the control layer. The physical system includes the pond environment, sensor nodes, and actuators. The cyber layer consists of data processing, predictive modeling, Digital Twin simulation, and a supervisory interface. The control layer executes validated decisions for aeration and feeding.

The system state at time (t) is defined as:

(1) $$X\left(t\right)={\left[DO\left(t\right),\;NH_{3}\left(t\right),\;Temp\left(t\right),pH\left(t\right),Turb\left(t\right),A_{aer}\left(t\right),A_{feed}\left(t\right),E\left(t\right)\right]}^{T}$$

where *DO* denotes dissolved oxygen (mg/L), $NH_{3}$ ammonia concentration (mg/L), *Temp* water temperature (°C), pH acidity–alkalinity, *Turb* turbidity (NTU), $A_{aer}$ aerator state, $A_{feed}$ feeder state, and *E* actuator-level energy consumption (Wh).

This state-space formulation ensures consistency with the predictive model input features and enables unified representation for sensing, prediction, Digital Twin simulation, and control decision-making.

The framework operates through the following stages:

1. State definition;
2. IoT sensing with multi-rate acquisition (1 min for anomaly detection, 5 min for modeling)
3. Data preprocessing;
4. Predictive modeling;
5. Digital Twin validation as a physics-informed gatekeeping layer;
6. CPS control execution;
7. Feedback update.

Figure 1 illustrates the complete system architecture, where data flow (sensor-to-cloud-to-model) and control flow (validated actions to actuators) are explicitly distinguished. The Digital Twin serves as an intermediate validation layer that bridges the gap between data-driven prediction and physically interpretable control, thereby improving system reliability and explainability.

### 3.2. Data Acquisition and Processing (Perceptron Layer)

A distributed IoT sensing network monitors dissolved oxygen, ammonia, temperature, pH, turbidity, actuator statuses, and energy consumption and raw sensor data are acquired at 1-min intervals to ensure high responsiveness and anomaly detection. Data are consolidated into 5-min intervals for predictive modeling and Digital Twin simulation. This dual-rate approach harmonizes immediate responsiveness with computational efficiency.

The preprocessing pipeline comprises:

- Moving-average filtering;
- Linear interpolation for missing values;
- Timestamp synchronization;
- Min–max normalization.

Energy consumption is consistently expressed in watt-hour (Wh). Instantaneous power (W) is integrated over time:

(2) $$E\left(Wh\right)=P(W)\times t\left(h\right)$$

where *E* is energy consumption (Wh), *P* is power (W), and *t* is time (h).

While raw data are collected at 1-min intervals, all predictive modeling and Digital Twin simulations use 5-min aggregated data to ensure consistency across system layers. Sensor readings are periodically validated against calibration standards to reduce drift effects, particularly for ammonia and pH sensors.

The experimental setup was implemented as a pilot-scale, low-power ornamental aquaculture system with an approximate water volume of 200 L. To evaluate the effectiveness of the proposed energy-aware predictive control framework under resource-constrained conditions, low-power actuators, including a 5 W aerator (Resun, Shenzhen, China) and an 8 W water circulation pump (SOBO, Zhongshan, China), were utilized. This configuration enabled the assessment of Digital Twin-assisted predictive control under small-scale operational conditions with limited energy demand. Because the actuator loads operated under low-power conditions, the PZEM-004T module was calibrated and validated in the low-current operating range before experimental deployment.

#### Sensor Calibration and Validation

Prior to deployment, all environmental sensors were calibrated against commercially calibrated reference instruments to ensure measurement accuracy and reduce sensor drift effects. Specific sensor models and accuracy ranges are summarized in Table 1. Dissolved oxygen (DO) measurements were validated using a portable dissolved oxygen meter, while pH sensors were calibrated using standard buffer solutions (pH 4.0, 7.0, and 10.0). Ammonia (NH_3_) concentrations were periodically cross-checked using a commercial colorimetric water quality test kit. Validation results demonstrated strong agreement between the IoT sensing system and reference instruments. The DO sensor achieved an R^2^ value of 0.97 with a mean absolute error (MAE) of 0.18 mg/L. Although the NH_3_ sensor exhibited moderate drift during prolonged operation, periodic recalibration at 7-day intervals maintained a reliable R^2^ value of 0.91 throughout the deployment period.

### 3.3. Predictive Modeling in the Cyber Layer

To predict short-term changes in the environment, we use a Long Short-Term Memory (LSTM) network.

The model architecture consists of:

- Two stacked LSTM layers (64 units each);
- Dropout layer (rate = 0.2);
- Fully connected output layer.

The input tensor is defined as:

(3) $$X\in R^{B\times 24\times 8}$$

where *B* denotes the batch size, 24 represents the sequence length, and 8 corresponds to the number of input features, including environmental variables and actuator states.

The above represents 24 time steps (2 h at 5-min resolution) and 8 input features.

Training configuration:

- Optimizer: Adam;
- Learning rate: 0.001;
- Batch size: 32;
- Loss function: Mean Squared Error (MSE);
- Early stopping based on validation loss.

The predictive model incorporates multiple environmental and operational variables, as summarized in Table 2. The model generates one-step-ahead estimates for dissolved oxygen (DO) and ammonia (NH_3_), which are primary control variables and LSTM is selected due to its ability to capture temporal dependencies in relatively small datasets. Compared to more complex architectures such as Transformers, LSTM provides a better trade-off between prediction accuracy and computational efficiency for real-time deployment.

The predictive model does not directly control actuators. Instead, its outputs are passed to the Digital Twin validation layer to ensure safe and reliable decision-making.

### 3.4. Digital Twin-Assisted State Simulation

The Digital Twin integrates real-time data with physics-informed relationships to support scenario-based evaluation.

(4) $$\frac{d[DO]}{dt}=k_{L}a\left(DO_{sat}-DO\right)-R_{resp}-R_{nit}$$

where $k_{L}a$ is the oxygen transfer coefficient, representing the rate of oxygen transfer from air to water; $DO_{sat}$ is the saturation concentration of dissolved oxygen at a given temperature; $R_{resp}$ denotes the oxygen consumption rate due to respiration by fish and microorganisms; and $R_{nit}$ represents the oxygen consumption associated with the nitrification process.

(5) $$\frac{d[NH_{3}]}{dt}=I_{feed}-R_{nit}-R_{dil}$$

where $I_{feed}$ is the ammonia input rate resulting from feeding activities and fish excretion; $R_{nit}$ denotes the removal of ammonia through microbial nitrification; and $R_{dil}$ represents the dilution or removal rate due to water exchange or filtration processes.

These parameters collectively describe the dominant oxygen transfer and nitrogen transformation processes in small-scale aquaculture systems. All concentration-related variables, including DO and NH_3_, are expressed in mg/L, consistent with sensor measurements and predictive model inputs.

These equations are discretized using a forward Euler method:

(6) $$DO\left(t+1\right)=DO\left(t\right)+∆t[k_{La}\left(DO_{sat}-DO\left(t\right)\right)-R_{resp}-R_{nit}]$$

(7) $$NH_{3}\left(t+1\right)=NH_{3}\left(t\right)+∆t[I_{feed}-R_{nit}-R_{dil}]$$

where Δt = 5 min.

Model parameters are calibrated using least-squares fitting during an initial baseline period under manual operation and the calibrated parameters are subsequently validated by comparing simulated trajectories with observed sensor measurements.

Although simplified, these equations capture the dominant dynamics affecting water quality in small-scale ponds. The Digital Twin is not intended to fully replicate complex aquatic ecosystems but serves as a physically interpretable validation layer that improves decision reliability compared to purely data-driven approaches.

A simplified sensitivity analysis was conducted by independently varying key parameters, including the dissolved oxygen threshold, ammonia threshold, and oxygen transfer coefficient ($k_{L}a$), by ±10% to evaluate the robustness of the proposed control framework under moderate parameter uncertainty.

### 3.5. CPS-Based Control Strategy and Energy Optimization

The CPS layer integrates prediction, simulation, and control. The CPS-based predictive control procedure with Digital Twin validation is summarized in Algorithm 1.
**Algorithm 1.** CPS-Based Predictive Control with Digital Twin ValidationInput: $\mathrm{Predicted}\;\mathrm{dissolved}\;\mathrm{oxygen}\;DO_{pred}\left(t+1\right)$ $\mathrm{Predicted}\;\mathrm{ammonia}\;NH_{3}^{pred}\left(t+1\right)$ Output: Control for aerator and feeder 1. Initialize control thresholds: $\;\;\;\;\mathrm{Set}\;DO_{th}$ = 5 mg/L, $NH_{3}^{th}$= 0.5 mg/L 2. Evaluate predicted environmental state: $\;\;\;\;\mathrm{If}\;DO_{pred}\left(t+1\right)<\;DO_{th}$ then         Set candidate action: Activate aerator $\;\;\;\;\mathrm{If}\;NH_{3}^{pred}\left(t+1\right)<\;NH_{3}^{th}$ then         Set candidate action: Delay or reduce feeding 3. Digital Twin validation:      Simulate candidate actions using the Digital Twin model $\;\;\;\;\mathrm{Evaluate}\;\mathrm{resulting}\;\;DO_{sim}\left(t+1\right)$$,\;NH_{3}^{sim}\left(t+1\right)$ 4. Safety check:      If simulated states violate safety constraints, then        Apply fallback strategy (rule-based control) 5. Execute control action:      Apply validated actions to actuators 6. Feedback update:      Acquire new sensor data and update system state

The control thresholds are defined based on aquaculture water quality standards. Dissolved oxygen (DO) threshold is set to 5 mg/L, while ammonia (NH_3_) threshold is set to 0.5 mg/L.

To mitigate the effect of prediction uncertainty, all control actions are validated through the Digital Twin before execution. This validation layer reduces the risk of unsafe actuator behavior.

Energy optimization is achieved through:

- Adaptive scheduling;
- Duty cycling;
- Predictive load balancing.

### 3.6. Feedback, Adaptation, and Safety Mechanism

The system operates as a continuous closed-loop structure:

Sensor → Model → Digital Twin → Control → Feedback

Adaptation is achieved through periodic recalibration rather than continuous retraining, ensuring system stability and preventing overfitting. A fail-safe mechanism is implemented at the edge layer. In case of communication failure or excessive latency, the system reverts to predefined threshold-based local control rules.

The system is designed to operate under near real-time conditions, with an acceptable control latency of 1–2 s. If this threshold is exceeded, fallback control is activated.

The proposed framework is designed to support future extension toward multi-pond aquaculture environments, although the present study focuses on pilot-scale validation under a single-pond deployment scenario, with computational requirements primarily determined by data sampling frequency and prediction intervals.

Overall, this methodology provides a transparent integration of sensing, prediction, physics-informed simulation, and control, enabling reliable and energy-aware aquaculture management under real-world conditions.

### 3.7. Statistical Analysis

To statistically validate the observed reduction in energy consumption, daily energy consumption values obtained under manual operation and smart predictive control modes were compared using an independent samples *t*-test. Statistical significance was evaluated at a 95% confidence level (*p* < 0.05). Mean and standard deviation values were calculated to assess operational variability throughout the 45-day deployment period.

To reduce temporal autocorrelation effects associated with high-frequency IoT sampling, statistical analysis was conducted using daily aggregated energy consumption data rather than raw 1-min measurements. All statistical analyses were performed using Python 3.11 and associated statistical libraries.

## 4. Experimental Setup

**Figure 2:**
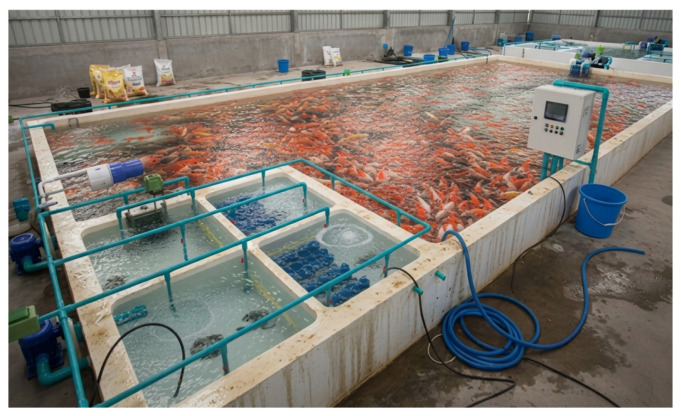
Experimental Setup of the Smart Koi Fish Farming System.

This section describes the experimental setup employed to implement and assess the proposed smart koi fish farming system under real-world situations. The experimental design corresponds with the predictive control framework outlined in Section 3, incorporating IoT sensing, Digital Twin (DT) simulation, deep learning prediction, Cyber-Physical System (CPS) control, and energy-efficient optimization (Figure 2).

The aim of the experiment is to assess system performance for environmental stability, prediction accuracy, and energy efficiency, while confirming that the noted enhancements are due to the proposed control approach rather than exogenous environmental fluctuations.

### 4.1. Hardware Infrastructure

A distributed IoT-based sensing and actuation infrastructure was deployed in an intensive koi aquaculture pond.

Environmental monitoring was performed using:

- DS18B20 digital temperature sensors;
- SEN0237 dissolved oxygen (DO) sensors;
- Gravity analog pH sensors;
- MQ-137 ammonia (NH_3_) sensors;
- turbidity sensors.

Energy consumption of actuators was measured using PZEM-004T single-phase energy metering modules.

All sensors were connected to NodeMCU ESP32 microcontrollers, which executed local data aggregation and transmitted data wirelessly to the cloud using IEEE 802.11 b/g/n Wi-Fi communication. Actuators, such as aerators, automatic feeders, and filtration pumps, were linked through relay modules and managed using the CPS framework. A Raspberry Pi 4 was utilized as an edge-computing unit to facilitate local buffering, initial processing, and fail-safe control during communication disruptions.

To ensure representative environmental sampling, sensors were submerged at a median depth of approximately 30 cm in a high-circulation zone, minimizing the influence of localized stagnant regions and capturing bulk water characteristics. To improve measurement reliability, selected parameters, particularly dissolved oxygen and ammonia, were periodically validated using reference-grade measurement instruments (e.g., laboratory-grade multi-parameter probes). These reference measurements served as ground truth for calibration and validation purposes.

The experimental setup was implemented as a pilot-scale ornamental aquaculture system with an approximate water volume of 200 L. To evaluate the effectiveness of the proposed energy-aware predictive control framework under resource-constrained conditions, low-power actuators, including a 5 W aerator and an 8 W water circulation pump, were utilized.

### 4.2. Software Architecture

The software architecture was developed to facilitate real-time monitoring, predictive analytics, Digital Twin synchronization, and closed-loop control, and sensor firmware was created using the Arduino IDE for reliable data acquisition and transmission. Incoming data streams were stored in a Firebase Real-Time Database and processed using a preprocessing pipeline that included:

- Noise filtering;
- Timestamp synchronization;
- Data validation.

Node-RED served as the primary integration platform, facilitating real-time visualization, execution of CPS control logic, and bidirectional communication between physical and cyber components, and the Digital Twin environment was established with Node-RED in conjunction with Python-based simulation modules, facilitating ongoing synchronization with real-time sensor data.

**Figure 3:**
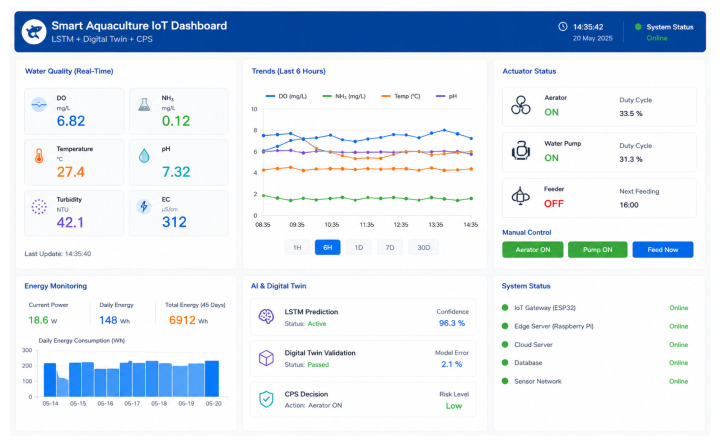
Real-time IoT dashboard for water quality monitoring and CPS-based predictive control.

LSTM-based predictive models were deployed in a cloud environment, and their outputs were forwarded to the Digital Twin and CPS layers for validation before control actions were executed. Figure 3 presents the real-time IoT dashboard used for water quality monitoring and CPS-based predictive control.

The dashboard interface enabled real-time monitoring of water quality parameters, actuator states, energy consumption, and Digital Twin validation results throughout the deployment period.

### 4.3. Data Collection and Experiment Execution

A continuous data collection program was executed over a 45-day period, including a broad spectrum of environmental and operational situations.

Sensor measurements included:

- Temperature (°C);
- dissolved oxygen (mg/L);
- pH;
- Turbidity (NTU);
- Ammonia concentration (mg/L);
- Actuator power consumption (W);
- derived energy consumption (Wh);
- Actuator ON/OFF states.

Raw data were collected at 1-min intervals to capture transitory fluctuations and subsequently consolidated into 5-min intervals for predictive modeling and control analysis.

Two operational modes were evaluated:

1. Baseline Mode: Conventional manual operation based on fixed schedules and human judgment.
2. Smart Control Mode: Autonomous CPS-driven operation guided by LSTM predictions and Digital Twin validation.

To provide an equitable comparison and mitigate bias from environmental fluctuation, the 45-day period was divided into alternating cycles of baseline and smart control modes, encompassing analogous diurnal temperature patterns, meteorological conditions, and operational settings. This approach facilitates the separation of algorithmic control effects from external environmental factors.

### 4.4. Digital Twin and Deep Learning Model Implementation

The Digital Twin was continuously synchronized with real-time sensor data, preserving a virtual depiction of the pond’s environmental and operational conditions.

The Digital Twin was utilized to:

- simulate candidate control strategies;
- evaluate risk–energy trade-offs;
- validate control actions prior to physical execution.

Simulations were conducted on scenarios including temporary aerator shutdowns during anticipated low-risk times to evaluate potential energy savings and environmental effects.

**Deep Learning Model Configuration**

The LSTM model was trained using:

- sequence length: 24 time steps (2 h at 5-min resolution);
- batch size: 32;
- epochs: 150;
- optimizer: Adam;
- loss function: Mean Squared Error (MSE).

To mitigate overfitting due to the relatively limited dataset duration, the following were applied during training:

- early stopping;
- validation-based monitoring.

**Ground Truth Validation and Calibration**

Selected sensor measurements were periodically validated against reference-grade instruments to ensure measurement accuracy and reduce sensor drift effects.

Digital Twin parameters (e.g., oxygen transfer coefficient $k_{L}a$, respiration rate, and nitrification terms) were calibrated using least-squares fitting during an initial baseline period. A calibration–validation loop was implemented to maintain alignment between simulated and observed system behavior throughout the experiment.

**Sensor Calibration and Validation**

Prior to field deployment, all environmental sensors were calibrated against commercially calibrated reference instruments to improve measurement reliability and reduce sensor drift effects. Dissolved oxygen measurements were validated using a portable laboratory-grade dissolved oxygen meter, while pH values were validated using a calibrated handheld pH meter. Ammonia measurements were periodically cross-checked using a commercial water quality test kit.

Calibration validation demonstrated strong agreement between the proposed sensing system and reference instruments. Dissolved oxygen measurements achieved an R^2^ value of 0.97 with a mean absolute error (MAE) of 0.18 mg/L, while ammonia measurements achieved an R^2^ value of 0.91 despite moderate drift during prolonged deployment. These procedures improved consistency between the physical pond and the Digital Twin simulation layer.

### 4.5. Experimental Procedure

The experiment began with system initialization and Digital Twin synchronization.

During operation:

- real-time sensor data were continuously collected and visualized;
- LSTM models generated rolling short-term forecasts;
- predicted values were evaluated against biological safety thresholds;
- candidate control actions were validated using the Digital Twin;
- validated actions were executed through the CPS framework.

Control decisions were therefore both predictive and simulation-validated, reducing the risk of unsafe or inefficient actuation.

Performance data from both baseline and smart control modes were recorded for comparative analysis.

### 4.6. Evaluation Metrics

System performance was evaluated across three dimensions:

- energy efficiency;
- environmental stability;
- predictive accuracy.

1. Functional Energy Breakdown

Energy consumption per module is defined as:

(8) $$E_{module,i}=P_{active,i}\times t_{active,i}$$

where

- $P_{active,i}$ is the average power consumption of module i (W);
- $t_{active,i}$ is the total operating time of module i (h);
- thus $E_{module,i}$ is expressed in watt-hour (Wh).

2. Event-Driven Energy Analysis

(9) $$∆E_{event}=E_{afterEvent}-E_{beforeEvent}$$

where $E_{afterEvent}$ and $E_{beforeEvent}$ are cumulative energy values measured before and after a specific event (Wh).

3. Time-Sliced Energy Analysis

(10) $${\bar{E}}_{slice}=\frac{1}{N_{slice}}\sum_{i=1}^{N_{slice}}E_{i}$$

where:

$N_{slice}\;$ is the number of observations within the time slice n.

$E_{i}\;$ is the energy consumption recorded at time step *i* (Wh).

By examining daily energy patterns, the system enables dynamic adjustment of operational strategies to reduce superfluous energy consumption during times of minimal environmental demand.

4. Mode-Based Energy Saving

Mode-Based Analysis compared the performance between the baseline manual operation mode and the smart CPS-controlled mode. The energy saving efficiency $\eta_{saving}$ was determined using:

(11) $$\eta_{saving}=(\frac{E_{baseline}-E_{smart}}{E_{baseline}})\times 100$$

where:

$E_{baseline}\;$ is the total energy consumption under manual control (Wh).

$E_{smart}$ is the total energy consumption under CPS-based smart control (Wh).

This investigation confirmed the efficacy of intelligent control systems in attaining significant energy reductions while maintaining biologically acceptable water quality conditions.

5. Predictive Accuracy

Predictive Load Forecasting assessed the precision of LSTM models in forecasting essential environmental factors. The evaluation of forecasting ability was conducted utilizing two statistical error metrics: Root Mean Squared Error (RMSE) and Mean Absolute Error (MAE), which are defined as follows:

(12) $$RMSE=\;\sqrt{\frac{1}{n}\sum_{i=1}^{n}{\left(y_{i}-\hat{y_{i}}\right)}^{2}}$$

(13) $$MAE=\frac{1}{n}\sum_{i=1}^{n}\left|y_{i\;}-\hat{y_{i}}\right|$$

RMSE is emphasized as a sensitivity metric to detect large prediction errors that may pose risks to fish welfare, while MAE provides an overall measure of average prediction bias.

**Uncertainty Consideration**

Energy reduction results are analyzed across multiple temporal segments to account for environmental variability and ensure that performance improvements are robust rather than condition-specific.

### 4.7. Statistical Significance Analysis

To statistically validate the observed reduction in energy consumption, daily energy consumption values obtained under manual operation and smart predictive control modes were compared using an independent samples *t*-test. Statistical significance was evaluated at a confidence level of 95% (*p* < 0.05). In addition, 95% confidence intervals (CI) were calculated to quantify uncertainty associated with the reported energy reduction.

To minimize bias caused by environmental variability, the statistical comparison was performed across alternating operational periods with comparable environmental conditions, including similar diurnal temperature patterns and weather conditions. All statistical analyses were conducted using Python statistical libraries.

## 5. The Experimental Results

The results from the 45-day implementation of the suggested smart aquaculture system are shown in this section and the evaluation concentrates on four dimensions: (i) IoT monitoring dependability, (ii) Digital Twin consistency, (iii) predictive model efficacy, and (iv) energy-aware control efficiency.

### 5.1. IoT Monitoring Performance

The IoT monitoring infrastructure demonstrated stable operation throughout the experimental period. A total of 12,960 records were expected at 5-min intervals over 45 days. Of these, 12,872 records were successfully received, corresponding to a transmission success rate of 99.3%.

The minimum percentage of lost data (0.7%) was mostly due to transient network congestion and intermittent cloud synchronization delays, which were concisely addressed during preprocessing, and latency analysis across 1000 samples showed an average delay of 231 ms, remaining below the 250 ms threshold. These results confirm reliable and near real-time data acquisition suitable for closed-loop control.

### 5.2. Digital Twin Consistency and Responsiveness

The Digital Twin consistently synchronized with real-time sensor data and was assessed on its capacity to replicate system dynamics.

The concordance between simulated and observed values was evaluated using the coefficient of determination R^2^.

- DO: R^2^ = 0.97;
- NH_3_: R^2^ = 0.94;
- Temperature: R^2^ = 0.91.

These results demonstrate a robust concordance between simulated and actual dynamics. The NH_3_ result is notably relevant, as ammonia dynamics are regulated by non-linear nitrogen cycle processes, encompassing food input, microbial nitrification, and water exchange. The Digital Twin’s capacity to consistently monitor NH_3_ fluctuations (R^2^ = 0.94) illustrates the efficacy of the suggested physics-informed modeling methodology in representing intricate biological dynamics.

System responsiveness analysis showed an average synchronization latency of 231 ms, with a maximum delay below 5 s. During the 45-day deployment, fallback control was triggered 11 times due to temporary communication latency exceeding the predefined threshold. In all cases, local threshold-based edge control maintained stable actuator operation without critical water quality deviation. The Digital Twin also enabled scenario-based validation, allowing control actions to be evaluated before execution, thereby reducing operational risk.

### 5.3. Predictive Performance of the LSTM Model

The LSTM model was trained on multivariate time-series data and evaluated on a 20% holdout test set.

As summarized in Table 3, the LSTM model achieved lower RMSE and MAE values compared with GRU and Simple RNN models across the evaluated environmental variables. These findings indicate that LSTM performs better in capturing temporal dependencies, particularly for DO and NH_3_, which exhibit nonlinear and delayed responses. RMSE was emphasized for its ability to penalize large prediction errors that could jeopardize fish welfare, whereas MAE reflects the average prediction bias. The predicted trends closely followed the observed diurnal patterns, enabling proactive control actions.

**Figure 4:**
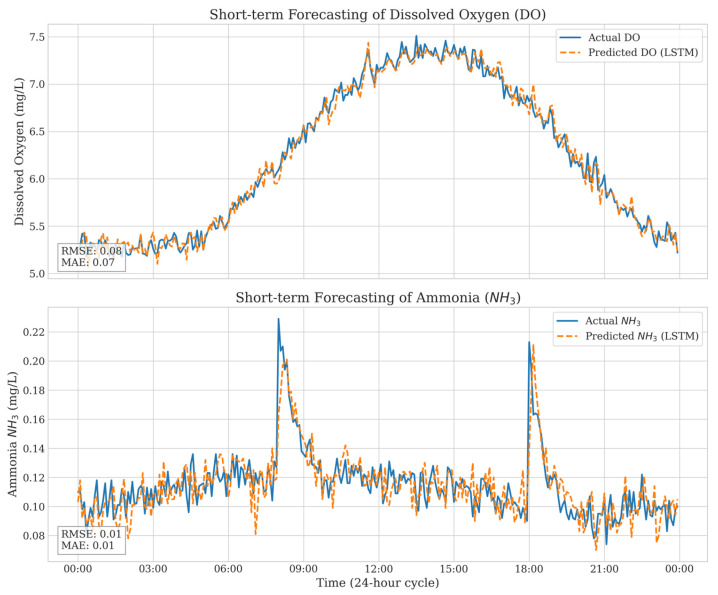
Actual vs. Predicted of Ammonia and Dissolved Oxygen.

Figure 4 illustrates the comparison between actual and LSTM-predicted dissolved oxygen and ammonia concentrations during the experimental period. The LSTM model successfully captured short-term environmental dynamics and closely followed the observed diurnal patterns of dissolved oxygen and ammonia fluctuations. The model accurately replicated rapid increases in ammonia levels associated with feeding events despite the nonlinear behavior of nitrogen cycling processes. Although slight deviations were observed during rapid environmental transitions, overall predictive consistency remained high. The low RMSE and MAE values further indicate that the proposed forecasting framework provides sufficiently reliable short-term predictions to support proactive control decisions within the Digital Twin-assisted CPS architecture.

### 5.4. Energy-Aware Control Performance

The integration of real-time monitoring, Digital Twin-based validation, and deep learning-driven forecasting facilitated the creation of an intelligent, energy-conscious control strategy for aquaculture operations and the system utilized predictive insights, namely for dissolved oxygen (DO) and ammonia (NH_3_), to dynamically modify actuator functions, such as aerator and feeder operations, enhancing energy efficiency while preserving biologically stable water conditions.

A comparative evaluation was conducted over a 45-day deployment period under two operational modes: (1) a baseline manual mode, where actuators were operated based on fixed schedules and human judgment, and (2) a smart predictive mode, in which control decisions were driven by LSTM-based forecasts and validated through a Digital Twin prior to execution.

Table 4 presents the energy and operational comparison between manual and smart predictive control modes. The results demonstrate a substantial reduction in energy consumption. Total energy usage decreased from 9450 Wh in manual mode to 6912 Wh in smart predictive mode, corresponding to a 26.86% reduction. Statistical analysis confirmed that the observed reduction in energy consumption was significant (*p* < 0.001). The calculated 95% confidence interval further demonstrated the consistency of the energy-saving performance across multiple operational periods, indicating that the reduction was unlikely to result from random environmental variation alone. This improvement was primarily achieved through predictive timing optimization, which allowed the system to align actuator operation with anticipated environmental demand rather than fixed schedules.

The system efficiently utilized natural environmental buffers, including oxygen recovery powered by photosynthesis during daylight hours. During these intervals, dissolved oxygen concentrations naturally rose, allowing the system to postpone or halt aeration without jeopardizing while maintaining biologically acceptable water quality conditions. Conversely, manual operation often resulted in excessive aeration due to cautious or habitual management methods.

The following evaluation indicated that the decrease in energy consumption was facilitated by three principal mechanisms: (i) adaptive aerator scheduling informed by projected dissolved oxygen dynamics, (ii) minimization of superfluous duty cycles through prompt identification of stable conditions, and (iii) feeding regulation predicated on forecasted ammonia accumulation and the system preemptively postponed feeding events when NH_3_ levels were anticipated to surpass safe limits, thus minimizing metabolic waste production and the ensuing energy requirement for water treatment.

These results confirm that transitioning from reactive to predictive control enables substantial improvements in both energy efficiency and operational effectiveness. Importantly, energy reduction was achieved without compromising environmental stability, indicating that predictive control can reconcile the trade-off between fish welfare and operational cost.

The proposed methodology illustrates that Digital Twin-assisted predictive control can transition aquaculture management from experience-based practices to data-driven, energy-optimized decision-making, delivering a viable approach to sustainable aquaculture systems.

**Figure 5:**
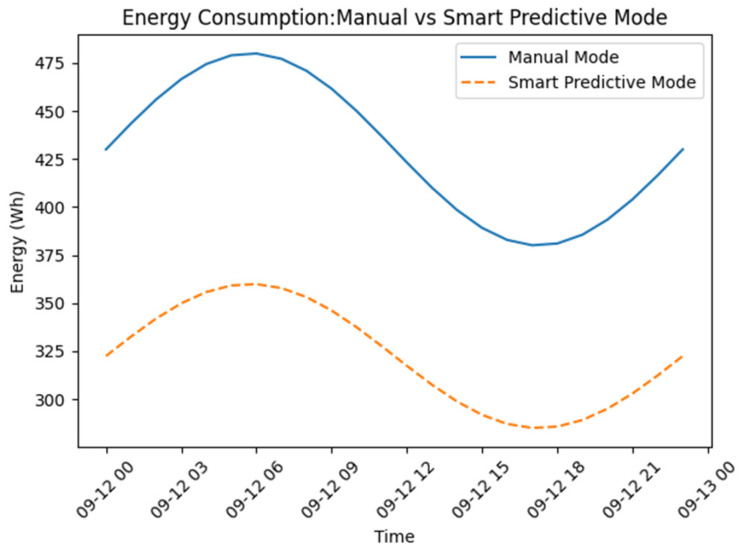
Energy per hour.

Figure 5 depicts the hourly energy consumption associated with manual and smart predictive control modes, thereby elucidating the temporal distribution of energy savings. As shown in Figure 5, the most significant reduction occurs during the afternoon period, where natural photosynthetic activity increases dissolved oxygen levels and the smart system exploits this environmental buffer by reducing unnecessary aeration, whereas the manual mode maintains relatively constant energy usage.

### 5.5. Closed-Loop Control and Energy Distribution

The effectiveness of the proposed closed-loop control architecture was further assessed using a comprehensive energy distribution analysis among system components and the energy contribution of each module was assessed utilizing Functional Energy Breakdown (Equation (8)), predicated on recorded power consumption and operational duration.

Table 5 shows that aeration remained the dominant actuator-level energy consumer, while the edge server also contributed substantially because it operated continuously throughout the observation period. In contrast, the combined computational overhead of the LSTM and Digital Twin modules remained below 1% of total energy consumption.

Table 5 presents the cumulative energy contribution of major system components during the 45-day smart predictive control deployment.

Energy consumption of sensing and edge computing components is relatively low in comparison to that of actuator modules, despite their continuous operation. This highlights that energy optimization in aquaculture systems should primarily focus on actuator-level control, especially aeration.

**Figure 6:**
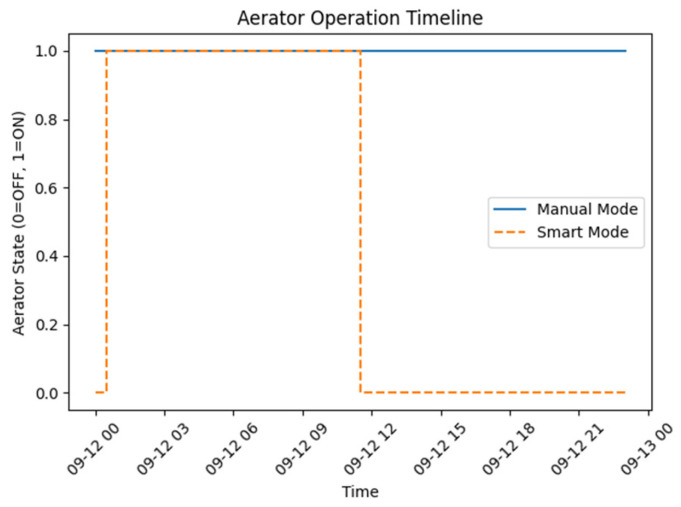
Aerator ON/OFF.

Figure 6 shows how the aerator works over time in both control modes and the duration of operation is reduced in comparison to the continuous operation observed in manual mode, as the smart predictive control dynamically modifies aerator activation based on predicted environmental conditions.

Event-driven analysis (Equation (9)) was employed to examine system responses to critical environmental events, such as feeding, dissolved oxygen depletion, and ammonia spikes. As shown in Table 6, the system demonstrates adaptive behavior by responding to environmental changes in a controlled manner.

These results show that the control system operates proactively rather than reactively. For example, oxygen levels are restored through the activation of aeration in advance during DO depletion events, and feeding is strategically delayed to prevent further water quality degradation during predicted ammonia accumulation conditions.

The time-sliced energy analysis (Equation (10)) explains the temporal distribution of energy usage. Table 7 illustrates that energy savings are particularly significant during the afternoon and early evening hours.

This behavior can be explained by predictive timing optimization combined with natural environmental dynamics and in daytime, heightened photosynthetic activity boosts natural oxygen generation, serving as an environmental buffer. The smart control system exploits this scenario by diminishing aerator operation while maintaining water quality. In contrast, manual operation typically maintains fixed aeration schedules, leading to unnecessary energy consumption.

Overall, the findings show that prediction, validation, and actuation are successfully combined into a single control loop by the suggested closed-loop CPS framework and this facilitates adaptive, context-sensitive energy management, wherein actuator functionality is dynamically synchronized with anticipated system conditions and natural environmental phenomena. As a result, the system realizes substantial energy conservation while ensuring environmental stability, hence enhancing the efficacy of predictive and Digital Twin-assisted aquaculture management.

### 5.6. Discussion of Prediction Uncertainty and System Behavior

The integration of prediction, simulation, and control facilitates a shift from reactive to proactive aquaculture management. A fundamental design characteristic of the proposed system is that LSTM predictions are not employed directly for actuation. However, they are validated through the Digital Twin, which functions as a constraint layer that is informed by physics.

This layered architecture prevents unsafe control operations due to forecast uncertainty and prediction mistakes are checked for biological restrictions before execution by the Digital Twin, enhancing system dependability and explainability.

This method effectively connects data-driven prediction with system behavior that can be understood in a physical way, which is a problem with deep learning models that are “black boxes”. Future work will include detailed time-series case analysis demonstrating prediction deviation, Digital Twin correction, and final control actions, further validating the robustness of the proposed framework.

A simplified sensitivity analysis was conducted to evaluate the robustness of the Digital Twin-assisted control framework. Key parameters, including dissolved oxygen threshold, ammonia threshold, and oxygen transfer coefficient ($k_{L}a$), were independently varied by ±10% while monitoring their influence on aerator duty cycle and energy consumption.

The analysis indicated that dissolved oxygen threshold settings exerted the strongest influence on actuator activation frequency and total energy consumption. In contrast, moderate perturbations in oxygen transfer parameters produced comparatively smaller variations in system behavior. Overall, the sensitivity analysis demonstrated that the proposed framework remained operationally stable under moderate parameter perturbations, indicating acceptable robustness for pilot-scale deployment.

## 6. Discussion

The experimental results indicate that the integration of IoT, Digital Twin (DT), Cyber-Physical Systems (CPS), and Deep Learning (DL) into a cohesive aquaculture framework is both technically viable and operationally efficient in real-world scenarios. In addition to quantitative performance indicators, the findings offer significant insights into system-level behavior, control dynamics, and practical deployment limitations.

A significant discovery is the viability of implementing LSTM-based prediction models on a resource-limited edge computing platform (Raspberry Pi 4). Notwithstanding restricted computing resources relative to cloud-based GPU settings, the model attained consistent predictive accuracy (RMSE = 0.48 mg/L for DO and 0.41 mg/L for NH_3_) which corroborates previous research [33,35] demonstrating that lightweight deep learning models may be efficiently utilized in edge-based IoT systems, especially in aquaculture settings where uninterrupted cloud access may be unreliable.

This study’s primary contribution is the integration of prediction, simulation, and control inside a closed-loop framework and the proposed framework incorporates a Digital Twin as a validation layer, in contrast to traditional systems that depend on direct threshold-based actuation. This facilitates a shift from reactive management to proactive and risk-conscious decision-making.

The methodology distinctly differentiates prediction uncertainty from control safety from a system design standpoint and the LSTM model generates probabilistic predictions of future environmental conditions, whereas the Digital Twin assesses the outcomes of proposed control actions through physics-informed correlations. This layered architecture alleviates the notorious black-box characteristic of deep learning models, as control decisions are not only reliant on obscure model outputs but are corroborated by interpretable physical restrictions. The Digital Twin serves as an explainability layer, along with the burgeoning trends in Explainable AI (XAI), where openness and trust in automated decision-making are paramount.

The results also underscore the significance of ammonia (NH_3_) modeling, which is influenced by the nonlinear dynamics of nitrogen cycle, encompassing feeding input, microbial conversion, and dilution effects. The robust correlation between Digital Twin outputs and observed NH_3_ dynamics (R^2^ = 0.94) is particularly noteworthy, given that ammonia is more challenging to measure and predict than dissolved oxygen and this discovery indicates that the amalgamation of streamlined physics-informed models with real-time data can proficiently represent intricate biogeochemical processes in small-scale aquaculture systems.

Energy reduction (26.86%) represents another key outcome, but its mechanism extends beyond simple actuator reduction and the results indicate that energy efficiency is achieved through predictive timing optimization, where control actions are aligned with natural environmental dynamics. In particular, time-sliced analysis shows that the largest energy savings occurred during afternoon periods. This can be attributed to increased photosynthetic activity, which naturally elevates dissolved oxygen levels, and under these circumstances, the algorithm minimizes aerator activity, but manual management frequently sustains superfluous aeration.

This behavior can be seen as the system utilizing natural environmental buffers, harnessing the inherent resilience of the aquatic ecosystem to diminish external energy input. Unlike manual operation, which cannot foresee these dynamics, the proposed framework adeptly “interprets” ecosystem recovery capacity and modifies actuator utilization accordingly. This underscores a significant benefit of predictive control in aquaculture, because environmental processes are included into the management strategy instead of being seen as disturbances.

The Digital Twin further enhances system reliability by enabling scenario-based validation of control actions before execution, and this is particularly important in biological systems, where incorrect decisions may rapidly degrade water quality and fish welfare. While previous DT implementations in aquaculture have primarily focused on monitoring and visualization [25,26], this study demonstrates its role as a decision-support and risk-mitigation mechanism within a closed-loop CPS architecture.

Notwithstanding these strengths, many drawbacks were identified. The mean system latency of 231 ms, with sporadic surges beyond 400 ms, could restrict utility in ultra-low-latency control contexts, aligning with previous IoT research on communication limitations [8,10].

Sensor reliability also remains a challenge. Ammonia sensors demonstrated increasing drift during extended deployment, while electrical conductivity (EC) values obtained indirectly from total dissolved solids (TDS) include estimation errors under fluctuating mineral conditions, and these concerns have been well documented in environmental sensing systems [21,22]. However, the presence of a continuous feedback loop in the CPS architecture partially mitigates these effects, as control decisions are repeatedly adjusted based on real-time observations, preventing the accumulation of large deviations that could compromise fish safety.

Future developments may further enhance system capability and vision-based fish monitoring can provide biological feedback signals, including fish activity and stress behavior, in addition to chemical water quality indicators. This would enable the Digital Twin to integrate both environmental and behavioral conditions, aligning more closely with the Precision Fish Farming (PFF) framework. Moreover, reinforcement learning-based control techniques may facilitate adaptive optimization throughout extended time frames, while the incorporation of external environmental data (e.g., weather forecasts) could enhance predictive robustness amid fluctuating climatic conditions.

Compared with conventional threshold-based aquaculture systems, which typically activate actuators only after environmental parameters exceed predefined safety limits, the proposed framework enables proactive environmental management through predictive analytics and Digital Twin validation. By anticipating environmental fluctuations before threshold violations occur, the system reduces unnecessary actuator operation while maintaining biologically acceptable water quality conditions. Although a full experimental comparison with threshold-based automation was beyond the scope of the present pilot-scale deployment, the proposed framework differs from conventional reactive control by incorporating predictive forecasting and Digital Twin-assisted validation before actuator execution.

Unlike manual or purely reactive systems, the proposed architecture integrates forecasting, simulation, and control within a unified closed-loop framework. This integration contributes to improved energy efficiency and operational adaptability under dynamically changing pond conditions.

Overall, this study demonstrates that combining predictive analytics, physics-informed simulation, and closed-loop control provides a practical pathway toward energy-efficient and biologically aware aquaculture management, extending beyond traditional monitoring-oriented systems.

Nevertheless, maintaining dissolved oxygen and ammonia concentrations within biologically acceptable ranges is widely recognized as essential for reducing physiological stress and supporting fish welfare in ornamental aquaculture systems. Although the proposed framework demonstrated stable environmental regulation and energy-aware operation, the present study primarily focused on environmental and operational performance rather than direct biological assessment. Biological indicators such as fish growth rate, feeding efficiency, stress biomarkers, or cortisol concentration were not measured during the deployment period. Future research should integrate physiological and behavioral indicators to provide a more comprehensive evaluation of fish welfare under predictive control conditions.

## 7. Conclusions and Future Work

This study presented an integrated smart aquaculture framework for koi fish farming, combining Internet of Things (IoT) sensing, Digital Twin (DT)-based modeling, deep learning prediction, and Cyber-Physical System (CPS)-based control to enable energy-aware and adaptive system operation.

The proposed framework enhances traditional monitoring-based aquaculture systems by implementing a closed-loop architecture that incorporates prediction, simulation, and control and the Digital Twin serves as a validation layer that connects data-driven predictions with physical system behavior, facilitating scenario-based assessments of control actions prior to implementation. This architecture improves system explainability and reliability by mitigating the opaque characteristics of deep learning models, ensuring that control decisions are corroborated with interpretable physical limitations.

Experimental results from a 45-day real-world deployment demonstrate that the system operates reliably under practical conditions and the LSTM model achieved stable predictive performance for key environmental variables, while the Digital Twin maintained strong synchronization with the physical pond. The integration of these components enabled proactive control strategies, resulting in a 26.86% (*p* < 0.001) reduction in total energy consumption compared to manual operation, without compromising biologically acceptable water quality conditions for ornamental aquaculture operation, and this energy reduction is primarily achieved through predictive timing optimization, where the system exploits natural environmental buffers, such as photosynthesis-driven dissolved oxygen recovery, to minimize unnecessary actuator operation.

Rather than introducing a fundamentally new architecture, this study provides an empirical and system-level validation of how Digital Twin-assisted CPS frameworks can improve both energy efficiency and environmental stability in small-scale aquaculture systems. The findings demonstrate that combining predictive intelligence with physics-informed simulation enables a more balanced trade-off between operational cost and biological safety.

However, several limitations remain and communication latency associated with cloud-based orchestration may limit performance in time-critical applications. Sensor drift, especially in ammonia measurements, generates uncertainty necessitating regular recalibration. The existing solution depends on offline model retraining, potentially constraining flexibility to prolonged environmental changes. In addition, the present study was conducted under a single pilot-scale deployment scenario involving one pond and one ornamental fish species over a 45-day period. Therefore, broader validation across multiple pond scales, species, and environmental conditions is still required.

Future work will concentrate on augmenting system intelligence and resilience through various avenues and the use of biological feedback signals, like fish behavior and activity patterns, can enhance chemical water quality indicators, providing a more thorough depiction of system status. Secondly, the advancement of autonomous online model adaptation methods may enable the predictive model and control policies to perpetually adjust to evolving environmental conditions. Third, enhancements in sensing precision and communication protocols are necessary to facilitate scalable and dependable implementation in various aquaculture environments.

Overall, this study demonstrates that the integration of IoT, Digital Twin, CPS, and Deep Learning provides a practical pathway toward energy-efficient and intelligent ornamental aquaculture management, with potential applicability to broader smart farming environments following further large-scale validation.

## Figures and Tables

**Table 1:** Sensor, Actuator, and Calibration Specifications.

Sensor	Model	Range	Unit	Accuracy	Sampling	Calibration
DO	SEN0237	0–20	mg/L	±0.2	1 min	7 days
pH	SEN0161-V2	0–14	–	±0.1	1 min	buffer
Temp	DS18B20	−55–125	°C	±0.5	1 min	monthly
Turbidity	SEN0189	0–1000	NTU	±5%	1 min	14 days
NH_3_	MQ-137	0–10	mg/L	±5%	1 min	7 days
Energy	PZEM-004T	—	Wh	±1%	1 min	Factory
Aerator	Mini Air Pump	5	W	Factory rated	-	N/A
Water Pump	DC Circulation Pump	8	W	Factory rated	-	N/A

**Table 2:** Predictive Model Variables.

Feature	Unit	Role	Type
DO	mg/L	Input/Target	Continuous
NH_3_	mg/L	Input/Target	Continuous
Temp	°C	Input	Continuous
pH	–	Input	Continuous
Turbidity	NTU	Input	Continuous
Aerator	0/1	Input	Binary
Feeder	0/1	Input	Binary
Energy	Wh	Input	Continuous

**Table 3:** Prediction Accuracy Comparison (Model Benchmarking).

Model	Variable	RMSE (mg/L)	MAE (mg/L)
LSTM	DO	0.48	0.34
	NH_3_	0.41	0.28
	Temp (°C)	0.35	0.23
GRU (baseline)	DO	0.55	0.39
	NH_3_	0.47	0.33
Simple RNN	DO	0.62	0.45
	NH_3_	0.53	0.37

**Table 4:** Energy and Operational Comparison: Manual Mode vs. Smart Predictive Mode (45 Days).

Metric	Manual Mode (45 Days)	Smart Control Mode (45 Days)	Reduction (%)
Total Energy (Wh)	9450	6912	26.86%
Aerator Duty Cycle (avg. %)	47.2%	33.5%	29.1%
Feeding Events (estimated total)	135 (3.0 × 45 days)	99 (2.2 × 45 days)	26.7%

Statistical analysis confirmed that the reduction in energy consumption was highly significant (*p* < 0.001).

**Table 5:** Functional Energy Breakdown under Smart Predictive Mode (45 Days).

Module	Average Power (W)	Active Time (h)	Energy (Wh)	Duty Cycle (%)
Water Pump	8.0	337.5	2700	31.3
Aerator	5.0	360	1800.0	33.3
Feeder	15.0	1.5	22.5	0.14
Edge Server	1.5	1080	1620	100.0
Sensor Network (ESP32 + sensors)	0.6	1080	648	100.0
LSTM & DT	1.0	121.5	121.5	11.25

**Table 6:** Event Response Results.

Event Type	Before Event (DO mg/L)	After Event (DO mg/L)	Before Event (Ammonia mg/L)	After Event (Ammonia mg/L)
Feeding	6.8	6.5	0.12	0.2
DO Dip	5.9	6.8	0.1	0.11
Ammonia Spike	7.2	6.9	0.3	0.45

**Table 7:** Time-Sliced Energy Results.

Time Slice	Average Energy Usage (Wh)
Morning (06:00–12:00)	38
Afternoon (12:00–18:00)	52

## Data Availability

The original contributions presented in this study are included in the article. Further inquiries can be directed to the corresponding authors.
